# Biomimetic osteochondral regeneration of the TMJ condyle: integrating BMP-2 osteoinduction, MSC-mediated fibrochondrogenesis, and gradient 3D-printed scaffolds

**DOI:** 10.1016/j.jobcr.2026.101501

**Published:** 2026-08-17

**Authors:** Bernardo Correia Lima, Michelle Alonso Coutinho, Pedro Américo Felizardo dos Santos, Ricardo Grillo, Mariana Aparecida Brozoski, Fernando Melhem-Elias

**Affiliations:** a Department of Oral and Maxillofacial Surgery, Traumatology and Prosthesis, Dental School of University of São Paulo, São Paulo, SP, Brazil; b School of Dentistry, Estácio de Sá University, Nova Iguaçu, RJ, Brazil; c Oral and Maxillofacial Surgeon, Sirio Libânes Hospital, São Paulo, SP, Brazil

**Keywords:** Temporomandibular joint, Condyle, Tissue engineering, Bone morphogenetic protein-2, Mesenchymal stem cells, 3D printing, Osteochondral regeneration

## Abstract

**Background:**

Biological and biomaterial-based approaches for temporomandibular joint (TMJ) condylar reconstruction have advanced substantially, yet no unified regenerative strategy currently reproduces the native osteochondral unit, particularly the fibrocartilage–bone hierarchy and its biomechanical demands.

**Methods:**

An integrative evidence synthesis was performed across four translational domains relevant to biomimetic condylar regeneration: (1) ultra-low-dose rhBMP-2 osteoinduction, (2) bilaminar osteochondral scaffolds, (3) MSC/TGF-β3–driven chondrogenesis, and (4) zonal or gradient 3D-printed constructs. Evidence was interpreted through mechanistic plausibility, scaffold design, safety constraints, and translational readiness for oral and maxillofacial surgery.

**Results:**

Convergent findings support the feasibility of biomimetic osteochondral regeneration. Controlled BMP-2 delivery enables potent osteoinduction when spatially confined and dose-restricted. Bilaminar constructs reproducibly generate stratified cartilage–bone organization and improve interface integrity. MSC/TGF-β3 approaches promote fibrocartilage-like matrix formation, particularly when combined with instructive scaffolds. Gradient 3D printing enhances anatomical fidelity and transitional biomechanics, improving construct coherence across zones.

**Conclusions:**

A multimodal regenerative framework integrating controlled osteoinduction, scaffold-based interface engineering, and biologically guided fibrocartilage regeneration may represent a promising translational pathway toward biologically engineered TMJ condylar reconstruction, although current evidence remains predominantly preclinical.

## Highlights

• Controlled BMP-2 release enhances TMJ subchondral bone repair safely.
• Bilaminar scaffolds replicate native TMJ osteochondral architecture.
• MSC and TGF-β3 signaling drive stable fibrocartilage tissue formation.
• Zonal 3D printing enables patient-specific, biomechanical TMJ condyles.
• Multimodal strategies offer a path to biological TMJ reconstruction.

## 1 Introduction

**Fig. 1:**
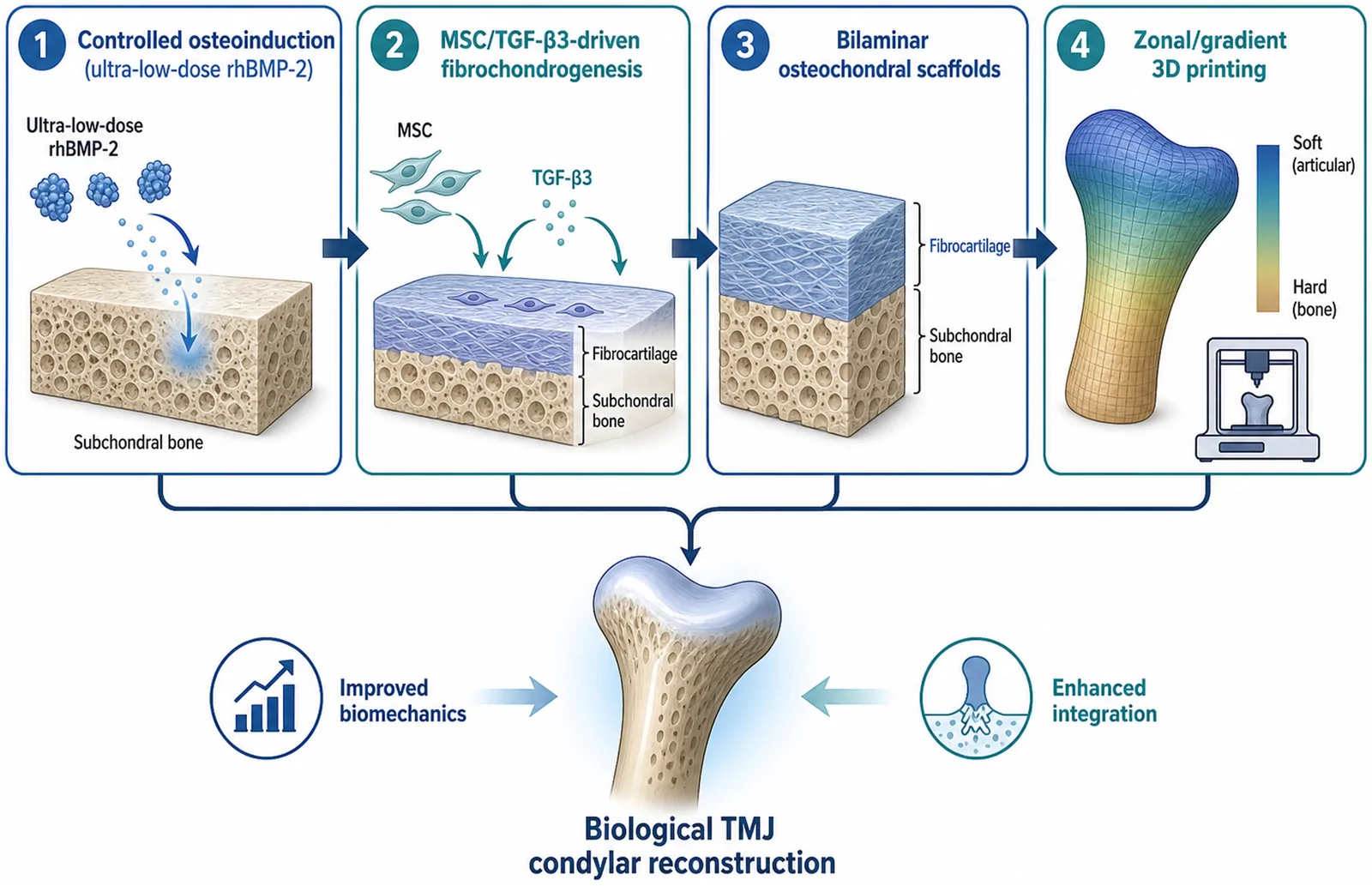
A multimodal regenerative strategy integrating controlled osteoinduction, MSC-driven chondrogenesis, bilaminar architecture, and zonal 3D printing offers the highest translational potential for biological TMJ condylar reconstruction.

Reconstruction of the temporomandibular joint (TMJ) condyle remains a persistent challenge in oral and maxillofacial surgery because the joint combines a fibrocartilaginous articular surface with subchondral bone, operates under multidirectional loading, and requires long-term functional durability. Conventional options, including costochondral grafts (CCG) and alloplastic total joint replacement (TMJR), can restore function, but are limited by donor-site morbidity, unpredictable remodeling and growth behavior, degenerative changes, and finite implant longevity. 1, 2, 3 These constraints continue to motivate biologically based alternatives capable of restoring both structure and function (see Fig. 1) (Fig. 1).

The need for improved condylar reconstruction is particularly acute in specific patient groups. Young patients with post-traumatic condylar defects or congenital deformities face decades of functional demand and a high likelihood of revision if treated with alloplastic devices. Patients with ankylosis, degenerative temporomandibular joint disease, or failed prior reconstructions may present with complex defects and compromised soft tissues, making conventional costochondral grafting or total joint replacement less predictable. Biological condylar reconstruction could theoretically offer more durable, growth-compatible, and patient-specific solutions for these challenging scenarios.

Biologic approaches to synovial joint disease support the rationale for regenerative strategies that integrate inflammation control with tissue regeneration. Evidence across joint models indicates that intra-articular biologic interventions can modulate the synovial microenvironment and influence cartilage homeostasis and repair pathways.^4^ In the TMJ context, orthobiologic injectables have shown clinically meaningful improvements in pain and function in TMJ osteoarthritis, and preclinical data similarly support cell-based and cell-derived approaches as chondroprotective and regenerative modalities.^5^^,^^6^

Within craniofacial regeneration, recombinant human bone morphogenetic protein-2 (rhBMP-2) has demonstrated strong osteoinductive capacity in mandibular reconstruction, enabling regeneration of critical-sized defects when appropriately delivered. 7, 8, 9, 10 Complementary craniofacial and dental tissue engineering studies have shown that controlled delivery of osteoinductive and angiogenic factors can enhance both bone formation and vascularization in alveolar and jawbone models. For example, immediate implant placement for alveolar ridge preservation has demonstrated that appropriate mechanical and biological conditions support favorable biomechanical and radiologic outcomes.^11^

In vitro and in vivo experiments using dental stem cells with strontium supplementation or vascular endothelial growth factor (VEGF)–loaded bioceramic and biphasic calcium phosphate scaffolds have further highlighted the importance of modulating osteoblastogenesis, osteoclastogenesis, and coupled angiogenesis–osteogenesis in optimizing bone regeneration. 12, 13, 14 These findings support the broader rationale for integrating controlled growth-factor delivery and angiogenic signaling into biomimetic approaches for TMJ condylar reconstruction. However, translation of BMP-2 signaling to the TMJ requires strict biological regulation. Mechanistic studies implicate dysregulated BMP activity in condylar cartilage degeneration, subchondral alterations, and ectopic mineralization through pathway-level perturbations, reinforcing the need for ultra-low-dose, spatially confined osteoinduction in this setting. 15, 16, 17.

Parallel advances in scaffold-based tissue engineering emphasize that a structurally competent platform is required to reproduce the native osteochondral hierarchy and maintain a stable cartilage–bone interface under functional loading. In mandibular and osteochondral models, cell-seeded and bioactive scaffolds have demonstrated improved integration and microarchitectural outcomes, supporting the concept that biological therapies are most effective when coupled to instructive biomaterials.^18^ Moreover, emerging additive manufacturing and computational design (CAD-CAM) approaches are enabling patient-specific constructs and graded architectures that better approximate the fibrocartilage-to-bone transition central to biomimetic condylar regeneration.^19^

Several prior reviews have addressed temporomandibular joint (TMJ) tissue engineering, BMP-2–based craniofacial regeneration, mesenchymal stem cell therapies, and 3D-printed scaffolds in isolation. However, these publications typically examine individual modalities or focus on general mandibular or osteochondral models rather than TMJ-specific osteochondral regeneration. In contrast, the present review integrates ultra-low-dose BMP-2 osteoinduction, bilaminar osteochondral scaffold architectures, MSC/TGF-β3–driven fibrochondrogenesis, and gradient 3D-printed constructs within a single translational framework specifically oriented toward biomimetic reconstruction of the TMJ condyle.

Accordingly, this integrative review examines the four convergent regenerative domains to clarify mechanistic rationale, safety constraints, and translational readiness, and to delineate a pragmatic framework for biologically engineered TMJ condylar reconstruction. This review is primarily translational in scope, integrating biological and mechanistic insights with scaffold design and preclinical evidence to inform future clinically oriented strategies rather than providing a comprehensive clinical guideline.

## 2 Methods

This review was conducted as an integrative synthesis of experimental, translational, and mechanistic evidence relevant to biomimetic reconstruction of the temporomandibular joint (TMJ) condyle. This approach was selected to allow conceptual integration across diverse preclinical models, biomaterial engineering strategies, and biologically driven regenerative therapies commonly investigated in oral and maxillofacial surgery.

### 2.1 Conceptual framework

Evidence was analyzed across four predefined regenerative domains selected for their direct relevance to TMJ condylar reconstruction and their demonstrated preclinical maturity: (1) ultra-low-dose rhBMP-2–mediated osteoinduction; (2) bilaminar or bilayer osteochondral scaffold architectures; (3) MSC- and TGF-β3–driven chondrogenesis and fibrocartilage regeneration; and (4) zonal or gradient 3D-printed osteochondral constructs. These domains reflect complementary biological and structural strategies required to replicate the native osteochondral unit of the TMJ.

### 2.2 Literature identification

Electronic searches were performed in PubMed/MEDLINE, Scopus, Web of Science, and Embase from database inception to February 2026, with the last search performed on March 2026. Search strategies combined controlled vocabulary and free-text terms related to temporomandibular joint and mandibular condyle (e.g., ‘temporomandibular joint’, ‘TMJ’, ‘mandibular condyle’), osteochondral or cartilage–bone regeneration (‘osteochondral’, ‘fibrocartilage’, ‘cartilage–bone interface’), and regenerative modalities (‘bone morphogenetic protein-2’, ‘BMP-2’, ‘mesenchymal stem cells’, ‘3D printing’, ‘bilaminar scaffold’, ‘gradient scaffold’). Search filters were not restricted by language or study design at the database level; non-English full texts were excluded at the screening stage when reliable translation was not feasible.

Both experimental investigations and high-quality narrative or systematic reviews were considered when they contributed mechanistic insight or translational relevance to TMJ reconstruction.

### 2.3 Eligibility criteria

Eligible studies included in vivo animal models, ex vivo tissue investigations, and in vitro experimental systems directly relevant to osteochondral regeneration of the TMJ, mandibular condyle, or anatomically analogous craniofacial osteochondral units. We included original experimental studies that evaluated at least one of the predefined domains (BMP-2 osteoinduction, bilaminar/biphasic scaffolds, MSC/TGF-β3–driven chondrogenesis, or zonal/gradient 3D-printed scaffolds), as well as high-quality narrative or systematic reviews that provided mechanistic or translational insight.

Non-TMJ models were included when they addressed mandibular or craniofacial osteochondral regeneration with clear potential applicability to TMJ condylar reconstruction; this limitation is explicitly noted in the synthesis where relevant. We excluded purely descriptive case reports, studies without osteochondral endpoints, and publications focusing solely on non-regenerative TMJ interventions (e.g., conservative management, arthroscopy, or conventional TMJ prostheses). Non-English articles were excluded if full-text translation could not be reliably obtained.

Two reviewers independently screened titles and abstracts for relevance and subsequently assessed full texts for inclusion. Disagreements were resolved by discussion and, when needed, consultation with a third reviewer. Because this was an integrative narrative review, no formal risk-of-bias tool was applied; instead, emphasis was placed on experimental design quality, anatomical relevance, and translational plausibility.

### 2.4 Data synthesis

Evidence was synthesized qualitatively, focusing on biological signaling pathways, scaffold architecture and interface stability, safety considerations, and translational feasibility. Rather than quantitative meta-analysis, emphasis was placed on cross-domain comparison and integration of biological and structural principles to support a translational framework for biomimetic TMJ condylar reconstruction. For each domain, we qualitatively graded translational readiness by considering anatomical relevance (TMJ-specific vs non-TMJ models), species and model size (small vs large animals), duration of follow-up, and integration of biomechanical or functional outcomes. These assessments are summarized in Table 2 as descriptive levels of evidence and translational maturity rather than formal GRADE ratings.

**Table 1:** Summary of experimental and translational evidence by regenerative domain for temporomandibular joint condylar reconstruction.

Domain	Study	Study design	Model/focus	Key findings
**Ultra-low-dose rhBMP-2 osteoinduction**	Suzuki et al., 2002	In vivo	Rabbit TMJ	rhBMP-2 enhanced fibrocartilage formation and subchondral bone repair
	Fan et al., 2017	In vivo	Rat mandible	Demonstrated strong dose-dependent osteoinductive response
	Fan et al., 2017 (TRB3)	Mechanistic	TRB3 signaling pathway	Amplification of BMP-2 signaling allowing dose reduction
	Khalil et al., 2025	In vivo	Supramolecular BMP-2 delivery	Sustained release reduced inflammation while preserving osteogenesis
	Yao et al., 2022	In vivo	Rat mandible	Improved angiogenesis–osteogenesis coupling
	Lyu and Lee, 2020	In vivo	Minipig mandible	BMP-2 hydrogel enhanced peri-implant bone quality
	Kawai et al., 2022	Gene transfer	Periodontal tissues	Localized BMP-2 gene delivery induced mineralized tissue formation
	Shen et al., 2022	In vivo	Calcium phosphate composite scaffold	Osteoclast-mediated remodeling yielded physiologic bone architecture
	Zhang et al., 2022	Mechanistic	TMJ osteoarthritis	Demonstrated PTHrP–BMP interaction in subchondral remodeling
	On, 2023	Review	Maxillofacial surgery	Highlighted clinical risks and dose sensitivity of BMP-2
**Bilaminar osteochondral scaffolds**	Nedrelow et al., 2023	In vivo	Goat TMJ	Patient-specific bilaminar scaffold achieved stratified regeneration
	Cao et al., 2021	In vivo	Osteochondral defect	Decellularized bilayer scaffold improved interface stability
	Chen et al., 2020	In vivo	Porcine TMJ	Autologous bilaminar graft restored condylar morphology
	Ma et al., 2025	Review	TMJ cartilage	Supported bilaminar scaffolds as biomimetic constructs
	Qian, 2025	Review	Orofacial scaffolds	Confirmed relevance of bilaminar scaffold design
**MSC and TGF-β3-driven chondrogenesis**	Li et al., 2015	Pathophysiology	TMJ synovial chondromatosis	Demonstrated potent TGF-β3-driven chondrogenesis
	Grayson et al., 2009	In vitro/in vivo	Craniofacial constructs	MSCs generated anatomically relevant bone and cartilage
	Choi et al., 2013	In vitro	Periodontal ligament MSCs	Strong upregulation of COL2A1 and aggrecan
	Du et al., 2023	Review	Cartilage biology	Identified TGF-β3 as regulator of lineage stability
	Jiang et al., 2024	Systematic review	TMJ	Confirmed preclinical efficacy of MSC-based therapies
	Bellinghen et al., 2018	Review	TMJ regenerative medicine	Highlighted MSCs as central biological component
	Fan et al., 2021	Review	TMJ fibrocartilage stem cells	Identified FCSCs as TMJ-specific cell source
**Zonal and gradient 3D-printed scaffolds**	Nedrelow et al., 2023	In vivo	Goat TMJ	Demonstrated anatomical fidelity and layered regeneration
	Golebiowska et al., 2024	In vivo	Osteochondral defect	ECM-zonal scaffold improved superficial cartilage
	Hu et al., 2023	Review	TMJ bioprinting	Summarized condyle and disc fabrication strategies
	Moosabeiki et al., 2024	Bioengineering	TMJ prosthesis	Functionally graded interfaces improved biomechanics
	Zhang et al., 2016	Review	Osteochondral tissue engineering	Demonstrated superiority of gradient scaffolds
	Atia et al., 2023	Review	Orofacial regeneration	Reviewed bioactive polymeric biomaterials
	Moura et al., 2020	In vitro	TMJ disc	Demonstrated feasibility of multi-material fabrication

**Abbreviations:** BMP-2, bone morphogenetic protein-2; ECM, extracellular matrix; MSC, mesenchymal stem cell; TMJ, temporomandibular joint; TRB3, tribbles pseudokinase 3.

**Table 2:** Biological mechanisms, principal limitations, and translational considerations of regenerative strategies for temporomandibular joint condylar reconstruction.

Regenerative strategy	Mechanism of action	Principal advantages	Main limitations and risks	Translational considerations
**Ultra-low-dose BMP-2 osteoinduction**	Activation of SMAD1/5/8 and Runx2 pathways promoting osteoblast differentiation and angiogenesis	Strong osteoinductive capacity; proven craniofacial efficacy; compatible with controlled-release systems	Risk of heterotopic ossification; inflammatory response with burst release; narrow therapeutic window in TMJ	Requires strict dose control and spatial confinement before clinical application
**Bilaminar osteochondral scaffolds**	Layered cartilage and bone matrices replicate native osteochondral hierarchy	High biomimicry; supports fibrocartilage and subchondral bone; favorable mechanical behavior	Variable interface integration; limited long-term load validation	Most advanced structural strategy; scalable manufacturing still required
**MSC and TGF-β3-driven chondrogenesis**	Directed MSC differentiation toward fibrocartilage lineage via TGF-β3 signaling	Induces fibrocartilage phenotype; immunomodulatory effects; accessible craniofacial cell sources	Risk of hypertrophic differentiation; requires microenvironmental regulation	Best applied in combination with biomimetic scaffolds
**Zonal and gradient 3D-printed constructs**	Spatial control of stiffness, porosity, and biochemical cues via multi-material printing	High anatomical fidelity; improved biomechanical transition; patient-specific design	Manufacturing complexity; limited TMJ-specific functional testing; regulatory uncertainty	Promising enabling technology; currently adjunctive rather than standalone

**Abbreviations:** BMP-2, bone morphogenetic protein-2; MSC, mesenchymal stem cell; SMAD, mothers against decapentaplegic homolog; TMJ, temporomandibular joint.

## 3 Results

The included studies were organized into four principal regenerative domains relevant to biomimetic reconstruction of the temporomandibular joint (TMJ) condyle. Additional mechanistic studies and high-quality reviews were used to provide cross-domain biological and biomaterial context.

### 3.1 Ultra-low-dose rhBMP-2–mediated osteoinduction

Early experimental evidence demonstrated that rhBMP-2 can modulate both subchondral bone repair and fibrocartilage organization within the TMJ. In a rabbit TMJ defect model, BMP-2 application resulted in improved fibrocartilage formation and enhanced chondrocyte alignment, supporting its role in osteochondral repair rather than isolated bone regeneration.^20^

In mandibular reconstruction models, BMP-2 consistently demonstrated potent, dose-dependent osteoinductive capacity. A seminal rat mandibular defect study confirmed robust bone formation following rhBMP-2 application, with micro-computed tomography and histologic analyses revealing rapid mineralization and defect bridging.^7^ These findings were corroborated by large-animal mandibular studies showing improved vascularization, structural integration, and mechanical stability when BMP-2 was incorporated into scaffold-based delivery systems.^7^^,^^9^^,^^10^

Mechanistic investigations focused on enhancing BMP-2 efficacy while minimizing adverse effects. Activation of the TRB3 signaling pathway amplified BMP-2–induced osteogenesis, enabling effective bone formation at reduced BMP-2 doses.^21^ This finding is particularly relevant to TMJ applications, where supraphysiologic osteoinductive signaling is associated with pathological remodeling.

Several studies addressed limitations related to burst-release and inflammatory responses associated with conventional BMP-2 carriers. Controlled-release hydrogels improved angiogenesis–osteogenesis coupling in mandibular defects,^22^ while supramolecular sustained-release systems attenuated early inflammatory reactions without compromising bone formation.^23^ In minipig models, hydrogel-based BMP-2 delivery around dental implants resulted in improved peri-implant bone quality and stability, supporting the translational relevance of controlled BMP-2 formulations.^24^

Alternative delivery strategies were also explored. BMP-2 gene transfer into periodontal and craniofacial tissues induced substantial mineralized tissue formation, suggesting a potential approach for spatially confined osteoinduction without exogenous protein administration.^25^ At the cellular level, BMP-2–calcium phosphate composite scaffolds promoted osteoclast-mediated remodeling, yielding bone microarchitecture more closely resembling physiologic condylar bone.^26^

However, mechanistic studies implicate dysregulated BMP activity in condylar cartilage degeneration, subchondral alterations, and osteochondral dysregulation through pathway-level perturbations, reinforcing the need for ultra-low-dose, spatially confined osteoinduction in this setting. 15, 16, 17.

Across craniofacial and mandibular models, BMP-2 efficacy appears to depend not only on absolute dose but also on spatial distribution and cumulative release over time. Studies employing sustained-release hydrogels and supramolecular carriers demonstrate that lower nominal doses can achieve comparable osteoinduction when release kinetics are tuned to provide a prolonged, submaximal stimulus rather than an initial burst.^22^^,^^23^ Although optimal in vivo concentrations vary between models and cannot be generalized, the consistent observation is that controlled, low-range exposure improves the balance between bone formation and adverse effects such as ectopic mineralization and inflammatory reactions.

### 3.2 Bilaminar osteochondral scaffold architectures

Bilaminar and biphasic scaffold designs emerged as the most biomimetic structural strategy for TMJ condylar regeneration. A patient-specific, 3D-printed polycaprolactone–hydroxyapatite (PCL–HA) condylar scaffold combined with a cartilage-matrix hydrogel demonstrated stratified osteochondral regeneration in a goat TMJ model, with early fibrocartilage formation and stable subchondral bone integration.^27^

Biomaterial-based bilaminar constructs incorporating decellularized cartilage over decalcified bone matrices achieved improved osteochondral organization and enhanced interface stability in osteochondral defect models.^28^ These scaffolds exhibited phase-specific porosity and stiffness, supporting differential cellular responses within cartilage and bone compartments.

Autologous cartilage–bone ramus–condyle grafts implanted in porcine models resulted in full-thickness fibrocartilage regeneration, restoration of condylar morphology, and long-term structural stability under functional loading.^29^ These findings provide strong proof-of-concept for bilaminar approaches that replicate the native osteochondral hierarchy of the TMJ.

Consistent conclusions across narrative and systematic reviews identified bilaminar scaffold architectures as the most biologically relevant platform for TMJ condylar reconstruction due to their ability to reproduce cartilage–bone stratification and mechanical transitions.^21^^,^^30^

### 3.3 MSC and TGF-β3–driven chondrogenesis

Multiple studies demonstrated that TGF-β3 is a potent regulator of chondrogenic and fibrocartilaginous differentiation within the TMJ environment. In TMJ synovial chondromatosis, TGF-β3 signaling drove proliferative chondrogenesis, highlighting its strong chondro-inductive capacity.^31^

Mesenchymal stem cells (MSC) derived from bone marrow, periodontal ligament, and TMJ-associated tissues exhibited robust osteochondral potential when exposed to chondrogenic cues. Human MSCs generated anatomically relevant craniofacial cartilage and bone constructs, supporting their applicability for condylar reconstruction.^32^ Periodontal ligament stem cells exposed to TGF-β3 demonstrated marked upregulation of type II collagen and aggrecan, consistent with fibrocartilage-like matrix formation.^33^

Mechanistic reviews identified TGF-β3 as a central regulator of cartilage lineage commitment, matrix synthesis, and hypertrophic control, emphasizing the importance of precise signaling modulation.^34^ Systematic reviews and experimental studies consistently supported MSC-based therapies as versatile biological components for TMJ regeneration, particularly when combined with biomaterial scaffolds providing structural and biochemical guidance.^35^^,^^36^

### 3.4 Zonal and gradient 3D-printed osteochondral constructs

Advances in additive manufacturing enabled the fabrication of anatomically accurate, patient-specific osteochondral constructs incorporating spatial gradients in stiffness, porosity, and material composition. Patient-fitted biphasic condylar scaffolds produced via 3D printing demonstrated layered tissue regeneration and improved morphological fidelity in large-animal TMJ models.^27^

Zonal scaffolds enriched with decellularized cartilage extracellular matrix promoted superior superficial cartilage formation compared with homogeneous constructs, underscoring the importance of zone-specific biochemical cues.^32^ Reviews of TMJ-specific additive manufacturing highlighted the growing use of stereolithography, extrusion bioprinting, and multi-material printing for condylar and disc regeneration.^37^

Functionally graded soft–hard interfaces and multi-material implants have been shown to improve load distribution and joint kinematics in experimental models, supporting the biomechanical relevance of gradient-based designs.^38^^,^^39^ Broader osteochondral tissue engineering literature demonstrates that graded or multi-layered constructs outperform monolithic designs in reproducing native transitional zones.^38^^,^^40^

Integrated biomaterial reviews emphasized the potential of multi-material and multiscale scaffold systems for orofacial regeneration, while acknowledging current limitations related to fabrication complexity, regulatory pathways, and long-term functional validation.^39^^,^^41^

Table 1 summarizes the pertinent evidence across each domain. A cross-domain synthesis of experimental outcomes, scaffold architectures, biological responses, and translational considerations is presented in Table 2.

## 4 Discussion

The progressive shift from monolithic biomaterials toward functionally graded and biologically instructive constructs represents a fundamental paradigm change in TMJ tissue engineering. The evidence synthesized in this review indicates that successful condylar reconstruction requires coordinated integration of biological signaling, cellular performance, and scaffold architecture, rather than reliance on a single regenerative modality.

### 4.1 Evidence base and conceptual proposals

This review synthesizes a heterogeneous body of predominantly preclinical evidence. Within each domain, we distinguish between findings supported by experimental data and conceptual proposals that have not yet been validated in TMJ-specific models. Statements describing in vivo outcomes in animal models or ex vivo tissue systems reflect established experimental evidence, whereas projections regarding ‘biological TMJR’ and long-term clinical performance should be interpreted as hypothesis-generating and subject to future verification.

### 4.2 Biological regulation of osteoinduction in the TMJ environment

From a historical perspective, BMP-2 delivery in craniofacial applications has evolved from simple absorbable collagen sponges toward more sophisticated carriers, including biphasic calcium phosphate scaffolds, polymeric hydrogels, microsphere-based systems, and gene-delivery approaches.^9^^,^^23^^,^^25^ These platforms aim to improve spatial confinement, modulate release kinetics, and integrate osteoinductive cues with structural support, reflecting a broader tissue engineering trend toward combination products that couple biologics with tailored biomaterial matrices.

Among osteoinductive agents, bone morphogenetic protein-2 (BMP-2) remains the most potent molecule for craniofacial bone regeneration. Experimental evidence demonstrates that BMP-2 can enhance subchondral bone repair and influence cartilage organization within TMJ defects, supporting its relevance beyond isolated osteogenesis.^7^^,^^10^^,^^20^ Mechanistic studies in the TMJ environment indicate that perturbations in BMP-related signaling pathways can drive pathological changes in cartilage and subchondral bone, suggesting a relatively narrow window between reparative and deleterious responses. 15, 16, 17.

Recent strategies have focused on mitigating these risks through ultra-low-dose delivery and spatial confinement. Sustained-release hydrogels, supramolecular carriers, and gene-transfer approaches demonstrated that effective osteogenesis can be achieved at substantially reduced BMP-2 concentrations while minimizing inflammatory responses. 22, 23, 24, 25 Mechanistic studies further indicate that interactions between BMP and TGF-β3 signaling pathways critically regulate osteochondral homeostasis within the TMJ, reinforcing the need for precise biological tuning rather than maximal osteoinductive stimulation.^31^^,^^34^ Collectively, these findings support the concept that BMP-2 is best utilized as a tightly regulated adjunct within multimodal regenerative strategies for TMJ reconstruction.

### 4.3 Structural importance of bilaminar osteochondral scaffolds

A recurring limitation of early tissue-engineered constructs was mechanical and biological failure at the cartilage–bone interface. Bilaminar and biphasic scaffold architectures directly address this limitation by replicating the native hierarchical organization of the TMJ condyle. Large-animal studies demonstrated that patient-specific bilaminar constructs support stratified fibrocartilage formation, stable subchondral bone regeneration, and improved interface integrity under functional loading.^27^^,^^29^

Decellularized cartilage and bone-derived extracellular matrix scaffolds further enhance osteochondral integration by providing tissue-specific biochemical cues and phase-appropriate mechanical properties.^28^ Consistent conclusions across narrative and systematic reviews indicate that bilaminar scaffold design currently represents the most biomimetic and structurally viable platform for TMJ condylar regeneration.^30^^,^^36^ These observations align with broader osteochondral tissue engineering principles emphasizing the importance of reproducing native tissue stratification.

### 4.4 Role of MSC-based fibrocartilage regeneration

While scaffold architecture provides structural guidance, biological functionality depends largely on cellular performance. Mesenchymal stem cells, particularly those derived from craniofacial tissues, demonstrate robust chondrogenic and immunomodulatory properties relevant to TMJ regeneration.^32^^,^^36^ TGF-β3 consistently emerged as a key regulator of fibrocartilage differentiation, inducing expression of type II collagen and aggrecan while supporting matrix organization characteristic of the TMJ articular surface.^31^^,^^34^

Despite these advantages, MSC-based strategies present potential limitations, including hypertrophic differentiation and ectopic chondrogenesis under prolonged or poorly regulated growth-factor exposure.^34^^,^^35^ These risks highlight the importance of combining MSC therapies with biomaterial systems that provide spatial control, mechanical cues, and controlled release of chondrogenic signals. When integrated within bilaminar or gradient scaffolds, MSC-based approaches demonstrate improved lineage stability and interface integration, supporting their role as a core biological component of multimodal TMJ regenerative strategies.^35^^,^^36^

### 4.5 Translational relevance of zonal and gradient 3D-printed constructs

Additive manufacturing technologies enable precise fabrication of patient-specific constructs with graded mechanical and biochemical properties. Zonal and gradient scaffolds demonstrate superior anatomical fidelity and biomechanical coherence compared with monolithic designs, particularly in reproducing the fibrocartilage-to-bone transition of the TMJ condyle.^27^^,^^32^^,^^37^ Functionally graded soft–hard interfaces further improve load distribution and mandibular kinematics in experimental models, supporting their relevance for joints subjected to complex multidirectional forces.^38^^,^^39^

Because the TMJ condyle operates under complex cyclic loading and coupled translational–rotational movements, long-term success of any regenerative construct will depend on its ability to maintain structural integrity and fibrocartilage–bone cohesion under physiologic mandibular function. Most current models do not reproduce the magnitude, directionality, or frequency of human masticatory forces, and only a minority of studies report quantitative biomechanical testing or kinematic analyses. Addressing this biomechanical gap will require dedicated large-animal TMJ models and standardized functional outcomes.

Nevertheless, the translational readiness of these technologies remains constrained by fabrication complexity, regulatory challenges, and the need for standardized manufacturing workflows (Atia et al., 2023; Moura et al., 2020).^39^^,^^41^ At present, gradient scaffold systems are best regarded as enabling platforms that enhance the performance of established biological strategies rather than stand-alone clinical solutions.

### 4.6 Regulatory and manufacturing considerations

Regulatory pathways for combination products that integrate living cells, growth factors, and patient-specific biomaterials remain complex and jurisdiction-dependent. Requirements for demonstrating safety, consistency of manufacturing, and long-term performance under real-world conditions are particularly stringent. Consequently, even technically feasible constructs may face substantial delays before clinical implementation, and early translational efforts will likely be confined to specialized centers with access to advanced GMP facilities and multidisciplinary regulatory expertise.

### 4.7 Translational challenges and future perspectives

Despite substantial experimental progress, several barriers remain before biological TMJ condylar reconstruction can be translated into routine clinical practice. Most available data derive from small-animal models that incompletely replicate the magnitude and directionality of human masticatory forces, underscoring the need for further large-animal and human cadaveric validation.^10^^,^^29^

Long-term biological stability also remains insufficiently characterized. The durability of MSC-derived fibrocartilage and the risk of late hypertrophy or ossification under chronic functional loading require continued investigation.^34^^,^^35^^,^^41^ Finally, regulatory considerations related to combination products incorporating cells, biologics, and advanced biomaterials represent additional translational hurdles that must be addressed through standardized protocols and scalable manufacturing strategies.^39^^,^^41^

A pragmatic translational roadmap for biomimetic TMJ condylar reconstruction would likely involve: (1) standardized in vitro optimization of growth-factor dosing, release kinetics, and scaffold architectures; (2) validation in small-animal models to screen safety and basic efficacy; (3) progression to large-animal TMJ models to evaluate anatomical fit, load-bearing capacity, and long-term durability; (4) development of good manufacturing practice (GMP)–compliant workflows for cell and scaffold fabrication; and (5) carefully monitored early-phase clinical trials in highly selected patients with limited alternatives. At each stage, comparative assessment against current standards of care, including costochondral grafts and alloplastic TMJ replacements, will be essential to justify further clinical translation.

## 5 Clinical applications

The translational framework proposed in this review suggests a paradigm shift from purely mechanical joint replacement to bio-integrated reconstruction. By leveraging the four regenerative domains, clinicians can potentially move toward a “biological TMJR” that offers several distinct advantages:

• Anatomical and Functional Fidelity: The use of CAD-CAM and 3D printing allows for the fabrication of patient-specific scaffolds that replicate the unique morphology of the mandibular condyle. Unlike standardized alloplastic implants, these constructs can be designed with zonal gradients in stiffness and porosity to better distribute complex, multidirectional masticatory loads. ^27^^,^^37^, ^38^, ^39^
• Controlled Tissue Hierarchy: The implementation of bilaminar scaffold architectures enables the simultaneous regeneration of subchondral bone and a fibrocartilaginous articular surface. This prevents the mechanical and biological failures often seen at the cartilage–bone interface in monolithic grafts. 28, 29, 30
• Precision Osteoinduction: By utilizing ultra-low-dose rhBMP-2 delivered via sustained-release hydrogels or supramolecular carriers, surgeons can achieve robust bone formation while minimizing the “collateral damage” of traditional BMP-2 applications, such as inflammatory edema and heterotopic ossification. ^9^^,^^22^, ^23^, ^24^
• Biological Longevity: MSC-based therapies, particularly those using TGF-β3-driven chondrogenesis, offer a pathway to restore the joint's natural reparative capacity. This approach could theoretically extend or improve functional longevity compared with current alloplastic materials and might reduce the need for complex revision surgeries in younger patients; however, such benefits remain speculative until confirmed in long-term clinical studies.^34^^,^^35^
• Pediatric and growing patients: Pediatric and growing patients represent a distinct subgroup in whom biological condylar reconstruction may have different risks and potential benefits compared with adults. Growth compatibility, modulation of condylar growth centers, and the long-term behavior of MSC-derived fibrocartilage under continuing skeletal development remain largely unexplored. While a biological construct that adapts to mandibular growth is conceptually attractive, the current evidence base does not yet support specific recommendations for children or adolescents, and any future trials in this population will require particularly stringent safety monitoring.^35^^,^^36^

While the convergence of these technologies is promising, several significant hurdles remain before biological condyle reconstruction can be considered a clinical standard of care:

• The BMP-2 ″Narrow Window”: Despite the efficacy of low-dose protocols, the TMJ remains uniquely sensitive to dysregulated BMP signaling. The risk of aberrant mineralization and subchondral sclerosis remains a primary concern, necessitating foolproof spatial confinement and dose-restriction protocols that are difficult to standardize across varying patient pathologies.
• MSC Lineage Stability: A persistent biological risk in stem cell therapy is hypertrophic differentiation. Ensuring that MSCs maintain a stable fibrocartilage phenotype under the chronic, high-magnitude loading of the human masticatory system, without progressing to late ossification, remains an area requiring longer-term validation.
• The “Masticatory Gap”: Much of the current evidence is derived from small-animal models (e.g., rats, rabbits) or large-animal models that do not fully replicate the magnitude and directionality of human forces. Consequently, the biomechanical durability of these bio-engineered constructs under human functional demands is still largely theoretical.
• Regulatory and Manufacturing Complexity: The transition to “combination products”, which integrate cells, bioactive proteins, and advanced biomaterials, faces a steep regulatory climb. The complexity of patient-specific manufacturing also raises questions regarding scalability, cost-effectiveness, and the standardization of workflows across different surgical centers.

Biological reconstruction of the temporomandibular joint condyle has progressed from isolated experimental concepts toward integrated, biomimetic strategies capable of addressing both structural and biological complexity. Evidence synthesized in this review indicates that successful regeneration depends on the coordinated application of controlled osteoinduction, biomimetic scaffold architecture, and biologically guided fibrocartilage formation rather than reliance on a single modality.

Ultra-low-dose BMP-2 delivery, bilaminar osteochondral scaffolds, MSC/TGF-β3–driven chondrogenesis, and zonal or gradient 3D-printed constructs represent complementary components of a translational regenerative framework, that will need continued large-animal validation, long-term functional assessment, and regulatory harmonization, essential before these approaches can be safely translated into routine clinical practice.

Importantly, these potential advantages are currently supported only by preclinical and conceptual evidence. No controlled clinical trials have yet demonstrated superior long-term outcomes of biological condylar reconstruction compared with established costochondral grafting or alloplastic TMJ replacement.

The evidence base synthesized here is highly heterogeneous with respect to species, defect models, scaffold materials, dosing regimens, and outcome measures. This heterogeneity, combined with the predominance of short-to mid-term follow-up and limited biomechanical testing under physiologic loading, constrains the reproducibility and generalizability of current findings. As a result, the translational conclusions of this review should be interpreted with caution, and no specific protocol can presently be recommended as a routine clinical standard. Until these gaps are addressed, biological TMJ condylar reconstruction should be regarded as an experimental and investigative avenue rather than a clinically validated alternative to established reconstructive options.

## Clinical relevance

This review synthesizes translational regenerative strategies for temporomandibular joint condylar reconstruction, highlighting biologically engineered alternatives to alloplastic joints through controlled osteoinduction, stem-cell–based fibrocartilage regeneration, and biomimetic scaffold design.

## Ethical approval

This article does not contain any studies with human participants or animals performed by any of the authors.

## Funding

This study was financed in part by the Coordenação de Aperfeiçoamento de Pessoal de Nível Superior -Brasil (CAPES) - Finance Code 001.

## Declaration of competing interest

The authors declare that they have no known competing financial interests or personal relationships that could have appeared to influence the work reported in this paper.
